# A New Approach for Super Resolution Object Detection Using an Image Slicing Algorithm and the Segment Anything Model

**DOI:** 10.3390/s24144526

**Published:** 2024-07-12

**Authors:** Muhammed Telçeken, Devrim Akgun, Sezgin Kacar, Bunyamin Bingol

**Affiliations:** 1 Computer Engineering, Institute of Natural Sciences, Sakarya University, Sakarya 54050, Turkey; 2 Computer Engineering, Sakarya University of Applied Sciences, Sakarya 54050, Turkey; 3 Software Engineering Department, Sakarya University, Sakarya 54050, Turkey; dakgun@sakarya.edu.tr; 4 Electrical and Electronics Engineering Department, Sakarya University of Applied Sciences, Sakarya 54050, Turkey; skacar@subu.edu.tr (S.K.); bunyaminnbingol@gmail.com (B.B.)

**Keywords:** object detection, super resolution, YOLO, SAM, SRGAN, xView, VisDrone

## Abstract

Object detection in high resolution enables the identification and localization of objects for monitoring critical areas with precision. Although there have been improvements in object detection at high resolution, the variety of object scales, as well as the diversity of backgrounds and textures in high-resolution images, make it challenging for detectors to generalize successfully. This study introduces a new method for object detection in high-resolution images. The pre-processing stage of the method includes ISA and SAM to slice the input image and segment the objects in bounding boxes, respectively. In order to improve the resolution in the slices, the first layer of YOLO is designed as SRGAN. Thus, before applying YOLO detection, the resolution of the sliced images is increased to improve features. The proposed system is evaluated on xView and VisDrone datasets for object detection algorithms in satellite and aerial imagery contexts. The success of the algorithm is presented in four different YOLO architectures integrated with SRGAN. According to comparative evaluations, the proposed system with Yolov5 and Yolov8 produces the best results on xView and VisDrone datasets, respectively. Based on the comparisons with the literature, our proposed system produces better results.

## 1. Introduction

Object detection stands out as one of the fundamental tasks of computer vision and aims to detect specific objects [1]. The development of satellite and Uncrewed Aerial Vehicle (UAV) technologies has enabled the development of object detection algorithms for aerial images. Using satellite and UAV images in practical applications plays an important role in areas such as monitoring natural disasters, mapping urbanization, and monitoring human and vehicle movements [2]. However, there are difficulties in detecting objects in satellite and UAV images. In particular, differences in object scale can make detecting small objects difficult. Satellites and UAVs capture images at different scales, causing significant differences in object size. Although deep learning algorithms have the potential to overcome these challenges, differences in object scale can make the detection of small objects difficult. Additionally, image noise can prevent the extraction of unique features of objects [3]. The widespread use of satellites and UAVs and the difficulties in detecting objects from images are just a few of the complex problems we face with the advancement of technology [4]. More successful work can be done on important issues, such as security, urban planning, and natural disaster monitoring, by providing solutions to current challenges.

This study proposes a three-stage structure to increase the mean average precision (mAP) value in object detection. In the first stage, a pre-processing algorithm called the Image Slicing Algorithm (ISA) is recommended to prevent the resizing of images in studies carried out with existing object detection algorithms. Resizing images causes a loss of resolution and distinctiveness of objects. Although feature loss is a problem in detecting large objects, the loss of distinctive features of small objects makes the detection of objects much more difficult. The developed ISA removes the objects at the edges while slicing images as much as possible. The developed algorithm uses the Segment Anything Model (SAM) to check the label accuracy of objects after the slicing process. The SAM compares the labels with box information using masks estimated on the image. This process is performed by overlap analysis between segmentation masks and box information. In this way, the accuracy of the annotations is checked, and if necessary, the label information of the sliced images is corrected. After the pre-processing phase, images for object detection are trained with the Super-Resolution Object Detection (SROD) algorithm, which is a hybrid of Super-Resolution Generative Adversarial Network (SRGAN) and Look Once Only (YOLO) algorithms. The proposed system is examined using the xView satellite and VisDrone aerial image datasets. Experimental results show that the proposed approach significantly improves object detection performance.

## 2. Related Work

In recent years, many studies have been carried out to improve the mAP in object detection. Yang et al. [5] proposed QeryDet, a feature pyramid-based object detector, to reduce costly computations using high-resolution images or feature maps. Their method first estimates the positions of small objects on low-resolution features and then uses these positions to obtain accurate detection results using sparsely oriented high-resolution features. In the experiments on the COCO dataset, they observed that QueryDet increased the detection mAP by 1.0 and the mAP small value by 2.0 and increased the high-resolution inference speed by an average of three times. Liu et al. [6] proposed a new framework, Image Adaptive YOLO (IA-YOLO), to overcome the challenges of balancing image enhancement and object detection tasks. Their proposed framework provides a structure in which each image can be adaptively enhanced for better detection performance. It includes a differentiable image processing (DIP) module for adverse weather conditions. Thanks to end-to-end collaborative learning, IA-YOLO can learn a suitable DIP to improve the YOLO detector, whose parameters are estimated by a small convolutional neural network. They achieved more effective results in both foggy and low-light scenarios with the IA-YOLO method. Wan et al. [7] proposed the YOLOv5 algorithm, which uses a feature pyramid, multi-detection head strategy, and a hybrid attention module for object detection in high-resolution optical remote sensing images. Their method obtained a 2.2% higher mAP value compared to YOLOv5 and an 8.48% higher value than YOLOX according to the SIMD dataset. Ming et al. [8] proposed a Design Search and Orientation Prediction Detector (TIOE-Det) for high-quality oriented object detection in aerial images. With TIOE-Det, they improved the alignment between classification and localization subtasks using the posterior hierarchical alignment (PHA) tag to optimize the detection pipeline. Additionally, TIOE-Det develops a balanced alignment loss and adopts a progressive orientation estimation (POE) strategy to estimate the orientation of objects. Tian et al. [9] proposed KCFS-YOLOv5, which they developed based on YOLOv5, to overcome the difficulties in remote sensing images. KCFS-YOLOv5 integrated Coordinate Attention (CA) and Bidirectional Feature Pyramid Network (BiFPN) into traditional YOLOv5 by optimizing the initial clustering points using the K-means++ algorithm to obtain suitable anchor boxes. Additionally, they added a new small object detection head and used the SIoU Loss function to increase the detection sensitivity of small objects in KCFS-YOLOv5. Fang et al. [10] proposed MIMDet, which is based on Masked Image Modeling (MIM) for object detection and offers to adapt pre-trained Vanilla Vision Transformer (ViT) efficiently. Bosquet et al. [11] proposed a new data augmentation method to increase the mAP value in detecting small objects. The GAN-based object generator includes a pipeline that combines object segmentation, image inpainting, and image blending techniques. The critical component of the pipeline is a new GAN-based architecture called DS-GAN. Their experiments on UAVDT and iSAID datasets showed that this proposed method improved the accuracy value of the latest popular object detection algorithms by up to 11.9% AP. Zhang et al. [12] proposed SuperYOLO, an object detection model developed for Remote Sensing Imagery (RSI), by combining multimodal data and using assisted super-resolution (SR) learning. SuperYOLO improves small object detection using symmetric compact multimodal fusion (MF) and includes a simple and flexible SR branch to learn high-resolution (HR) feature representations that can distinguish from extensive backgrounds with low-resolution (LR) input. Their experimental studies on the VEDAI RS dataset achieved 75.09% accuracy with SuperYOLO. Akyon et al. [13] proposed the Slicing Aided Hyper (SAHI) approach to solve the small object detection problem on xViex and Visdrone datasets. Olamofe et al. [14] evaluated the performance of the YOLOv3 model on the xView dataset by performing data augmentation by changing the color channels of the object, adding pure noises to the object images, and applying contrast enhancement operations. Shen et al. [15] used the combination of convolutional neural network (CNN) and YOLOv4 model to improve the performance of accuracy and speed in geographic object detection in large-size aerial images, using the multi-volume YOLO v4 model that balances accuracy and speed, as well as slicing strategy and Trimmed Maximum to process sizeable remote sensing images. They proposed a method using the non-maximum suppression (NMS) algorithm. With the proposed method, they achieved high speed and accuracy on the DOTA and DOTAv2 datasets. Lin et al. [16] introduced STPM_SAHI, a model that detects small target forest fires. Using the Swin Transformer backbone network, they extracted the features of forest fires with a self-attention mechanism and obtained larger information areas with a PAFPN feature fusion network. Integrating the SAHI approach significantly increased the accuracy of detecting small target wildfires. Shen et al. [17] proposed a method that includes scanning satellite images, detecting cloud-covered floors, object detection with the MIOU-based YOLO v7-Tiny method, and filtering the results with the truncated NMS method. They successfully carried out their experimental work on the DOTA-CD dataset. The AIR-CD dataset they created was used to verify the method’s reliability, and they demonstrated the effectiveness of their proposed method with the successful results they obtained. Pereira et al. [18] proposed a new method combining the YOLOR-CSP architecture using the SAHI framework to detect fundus lesions. They achieved successful results in their study on the DDR dataset with their proposed method. Akshatha et al. [19] introduced the Manipal-UAV person detection dataset to improve the detection of small objects in aerial images. By providing a comparative analysis with existing datasets and algorithms, they aimed to set a new benchmark for advancements in small object detection. Wang et al. [20] proposed YOLOX_w, an improved YOLOX-X model for UAV aerial photography object detection. YOLOX_w performed training on pre-processed training data using the SAHI algorithm and data augmentation to improve the performance of detecting small objects. With YOLOX_w enhanced with additional features, such as a PAN network and ultra-lightweight subspace attention module (ULSAM), they achieved an 8% higher detection accuracy than the baseline YOLOX-X on the VisDrone and DIOR datasets. Zhang et al. [21] introduced a new adaptive slicing method called Adaptive SAHI (ASAHI). This reduces unnecessary computation by adapting the number of slices to the image resolution and provides improved accuracy and inference speed with Cluster-DIoU-NMS. Their experimental work showed that ASAHI performed well on the VisDrone and xView datasets, with a 0.9% increase in mAP50 and a 20–25% reduction in computation time. Muzammul et al. [22] proposed the integration of SAHI to improve the analysis of aerial imagery using the RT-DETR-X model.

Although the studies in the literature include slicing, improvements are still needed to prevent the loss of cropped objects. The proposed work reduces object loss dynamically with the overlapping approach, depending on the image size. In addition, the proposed system checks the determined objects with SAM after slicing and updating the labels. By increasing the image quality with SRGAN, small objects are improved, and the model performance is increased.

## 3. Materials and Methods

**Figure 1:**
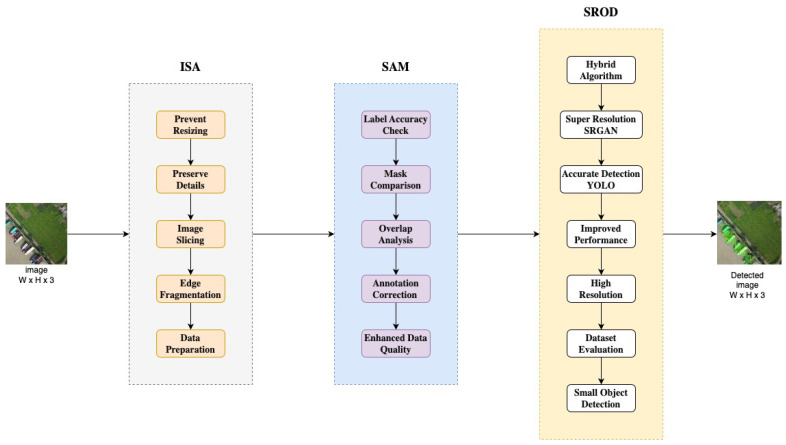
General flow diagram of the proposed system.

This study aims to improve the mAP value of object detection with the proposed methods. In the first stage, the Image Slicing Algorithm (ISA) is proposed to solve the image resizing problem. The Segmentation Anything Model (SAM) is used to ensure the accuracy of object labels after slicing. Image detection is processed using the Super-Resolution Object Detection (SROD) algorithm proposed as a hybrid structure. Experimental analyses are performed on the xView and VisDrone datasets. Figure 1 explains the main building blocks of the proposed system.

### 3.1. Datasets

It is thought that examining two different datasets for experimental studies is more suitable for performance evaluation. Datasets were specifically determined based on high-resolution, multi-class, and small object samples. When the literature was examined, it was seen that the xView and Visdrone datasets were more suitable for the specified criteria. In this study, experimental studies are carried out on these two datasets.

#### 3.1.1. xView Dataset

The xView [23] dataset contains satellite images covering different geographic regions and environmental conditions worldwide. This diversity of data is well-suited to evaluate and improve the overall performance of algorithms. The dataset contains over 1 million labels for more than 60 object classes. These object classes include vehicles, buildings, infrastructure elements, natural structures, and other objects. The dataset is designed for use in various fields, such as military, urban planning, disaster management, and environmental monitoring. See Table 1.

#### 3.1.2. VisDrone Dataset

The VisDrone [24] dataset was created to support computer vision tasks, such as object detection and tracking in images taken by uncrewed aerial vehicles (UAVs). It consists of images taken in various weather conditions, lighting conditions, and different environmental conditions, such as urban and rural. This diversity allows algorithms to be tested and developed in different scenarios. It contains high-resolution and detailed images. This allows small objects and complex scenes to be analyzed accurately. See Table 2.

### 3.2. Proposed Image Slicing Algorithm (ISA)

This study proposes the ISA as a data pre-processing algorithm. The proposed system prevents pixel losses in object detection studies with high-resolution images compared to existing technologies and potential application scenarios. This is provided by slicing the input image in accordance with the model input dimensions, which remains the same, and therefore, no pixel loss occurs. In the ISA, object labels in new images obtained by slicing are recalculated by proportioning them to the label bounding boxes in the original image. The purpose of proportioning the bounding boxes is to prevent the recording of erroneous border frames that may occur when the cropped objects are too small to represent the original object during slicing. The algorithm uses a dynamic slicing size structure that can be adjusted according to the image and model input dimensions to be used, focusing on preserving objects at their edge points and optimizing object labels. The equations of the slicing algorithm are shown below.

Let the crop size be C, while the width of the original image is represented by W and the height by H. The numbers of slices along the width ($n_{w}$) and height ($n_{h}$) are calculated in Equation (1).

(1) $$n_{w}=\frac{W}{C},\;n_{h}=\frac{W}{C}$$

The required overlapping amounts along the width ($p_{w}$) and height ($p_{h}$) are calculated in Equation (2).

(2) $$p_{w}=\frac{(n_{w}*C)-W}{n_{w}},\;p_{h}=\frac{(n_{h}*C)-W}{n_{h}}$$

The filled image dimensions are determined by Equation (3).

(3) $$W_{p}=W+p_{w},\;H_{p}=H+p_{h}$$

**Figure 2:**
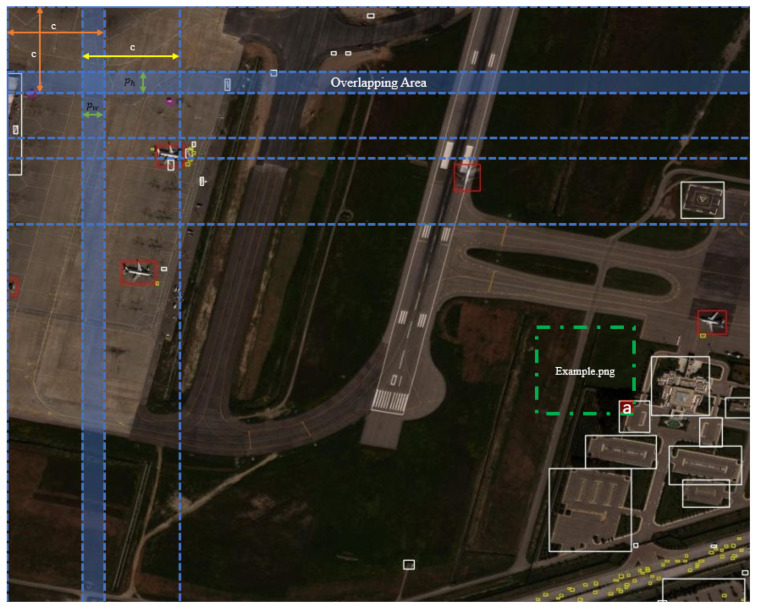
Example of applying the ISA structure on an image from the xView dataset. (a) The region to be untagged from the example.png image because it is smaller than 20% of the original tag size.

The original images are saved with tag files as new images divided into $n_{w}$ × $n_{h}$ slices. The visual of the application of the ISA algorithm on a sample image is seen in detail in Figure 2.

For the example image obtained by slicing, the label residue corresponding to point (a) is compared to the object label in the original image, and since the label residue at point (a) is less than 20 percent of the original label, the label residue at point (a) is compared to the example image. It is not saved as an object label. According to the content of the image, there is no object at the point that is not recorded as a label. Recording the labels by proportioning aims to remove the (a) point label, which is recorded as incorrect label information, and to prevent the models from learning incorrect information.

### 3.3. Segment Anything Model (SAM)

The Segment Anything Model (SAM) [25] is a deep learning model used in object segmentation. Segmentation separates certain objects in an image according to their boundaries [26]. The SAM uses advanced image processing and deep learning techniques to perform this segmentation process. The SAM extracts low- and high-level features from the image. These operations are generally performed using convolutional neural networks (CNNs). These features represent the position, shape, and other important details of objects in the image [27].

In object detection studies, the accuracy of the data labels and the fact that the objects in these labels fully reflect the distinctive features of the original object directly affect the success of the models. Checking the labels of the objects in the images obtained by slicing, verifying whether the objects in these labels fully reflect the original objects, and making the necessary adjustments improve the model performance. For this reason, the SAM was used in the system to verify the bounding boxes of objects in new images obtained by slicing and to update them when necessary. The SAM segments the object inside the bounding box. A segmentation mask is a binary image used to define the boundaries of the object. In the mask, object pixels are marked 1 (or 255), and background pixels are marked 0. The SAM verifies the object’s existence within the bounding box by analyzing the segmentation mask. A threshold value (t) is determined for the lower limit of the mask density. If the mask’s total value (density) is greater than the specified threshold value, the object is present. The bounding boxes of objects whose mask density is less than the threshold value are automatically deleted. The image to be processed is given to the SAM and prepared accordingly. The structure to be used for feature extraction is given in Equation (4).

(4) f(x)=Conv(x)

Here, *Conv* represents the convolution operations, and *f*(*x*) represents the extracted features. Equation (5) is used for the segmentation mask.

(5) mask=σ(Conv(f(x)))

Here, σ represents sigmoid or softmax activation functions. Finally, Equation (6) is realized according to the *t* threshold value determined to verify the objects.

(6) $$object_{true}=\sum \left(mask\right)>\;t$$

**Figure 3:**
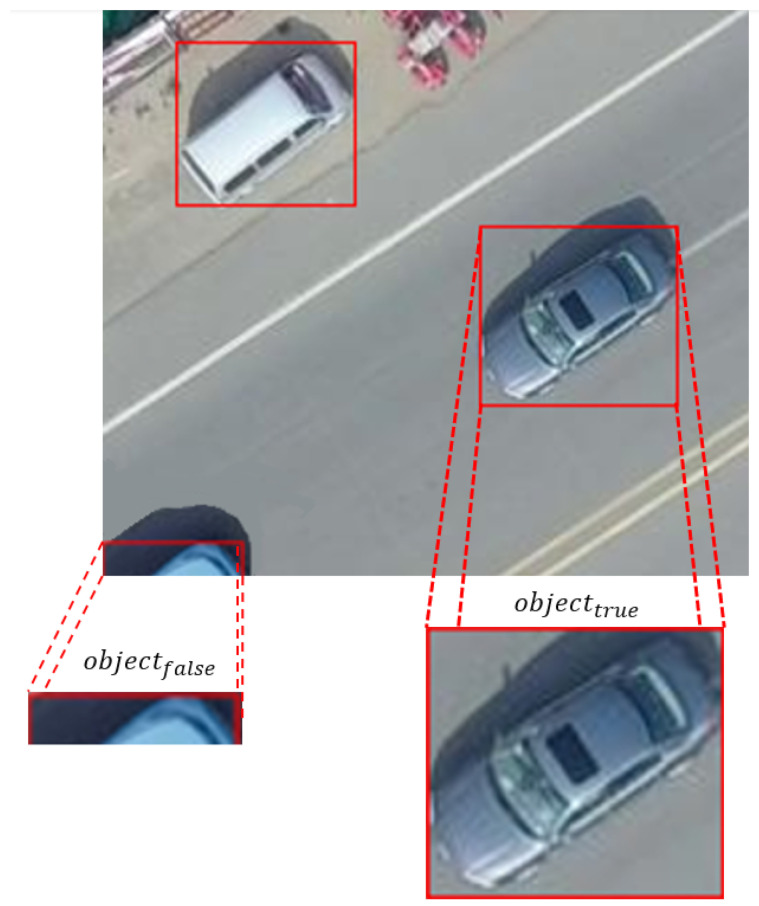
An example of checking the objects in the bounding box with the SAM for the image obtained by slicing from the VisDrone dataset.

The total intensity of the mask is calculated, and if it is greater than the threshold value, the object is considered present. Thanks to the SAM integrated into the system, high accuracy is achieved in the segmentation of objects, which allows the boundaries of objects to be determined more accurately. The existence of objects is automatically verified without the need for human intervention, which speeds up the process. In addition, this structure can be easily applied effectively to different image types and objects. The visual of controlling the objects within the boundary boxes with the SAM is seen in detail in Figure 3.

Since the object density value within the bounding box in the image (a) is greater than the specified threshold value *t*, $object_{true}$ is returned, and no changes are made to the label file for the bounding box. But since the object density in the bounding box (b) is less than the specified threshold value, $object_{false}$ is returned, and the coordinates of the bounding box (b) are deleted in the label file.

### 3.4. Proposed Super-Resolution Object Detection (SROD)

The Super-Resolution Object Detection (SROD) model is a hybrid structure obtained from Super-Resolution Generative Adversarial Network (SRGAN) [28] and Look Once Only (YOLO) [29] algorithms. The images pass through the SRGAN structure integrated into the YOLO architecture as soon as they enter the training model. By increasing the resolution of the images, the model learns the objects better. When training starts, the weights for the SRGAN model are loaded from a specified file.

The SRGAN model uses a deep learning model. The structure of the model is as follows:

**ResidualDenseBlock_5C Class.** This class defines a 5-layer residual dense block. Each layer has a convolutional layer (Conv2D) and a Leaky ReLU activation function.

**RRDB Class.** This class defines a residual dense block cluster consisting of three ResidualDenseBlock_5C blocks.

**RRDBNet Class:** This class defines a complete super-resolution model consisting of input and output layers, RRDB blocks, and various convolutional layers.

Convolutional layers are used to extract specific features by shifting a filter to the input image. This process can be seen in Equation (7):

(7) $$\left(W*I\right)\left(i,j\right)=\sum_{m=-k}^{k}\sum_{n=-k}^{k}W\left(m,n\right).\left(i+m,j+n\right)$$

Here, W represents the filter, I represents the input image, and * represents the convolution operation. (*i*, *j*) are the coordinates of the filtered image. Leaky ReLU is a non-linear activation function and is defined in Equation (8).

(8) $$f\left(x\right)=\left\{\begin{array}{ccc} x & if & x>0\\ \alpha x & if & x\leq 0 \end{array}\right.$$

Here, α is a small positive constant for the negative parts. Residual blocks facilitate learning by adding the input to the output and are calculated as in Equation (9).

(9) $$y=F(x,W_{i})+x$$

Here, *x* represents the block input, and *F* (*x*, $W_{i}$) represents the output calculated by convolutional layers and activation functions. Dense blocks enrich information by connecting the outputs of all intermediate layers. The dense block structure can be seen in Equation (10).

(10) $$x_{l+1}=H\left(\left[x_{0},x_{0},\ldots \ldots x_{l}\right]\right)$$

Here, [*x*${\;}_{0}$, *x*${\;}_{1}$, ……*x*${\;}_{l}$ ] refers to the combining of the outputs of all previous layers, and *H*${\;}_{l}$ refers to the 1st layer operations. Upsampling is used to make the image high-resolution. This process is usually done by nearest neighbor interpolation and is expressed in Equation (11):

(11) $$y\left(i,j\right)=x\left(\left\lceil \frac{i}{s}\right\rceil ,\left\lceil \frac{i}{s}\right\rceil \right)$$

**Figure 4:**
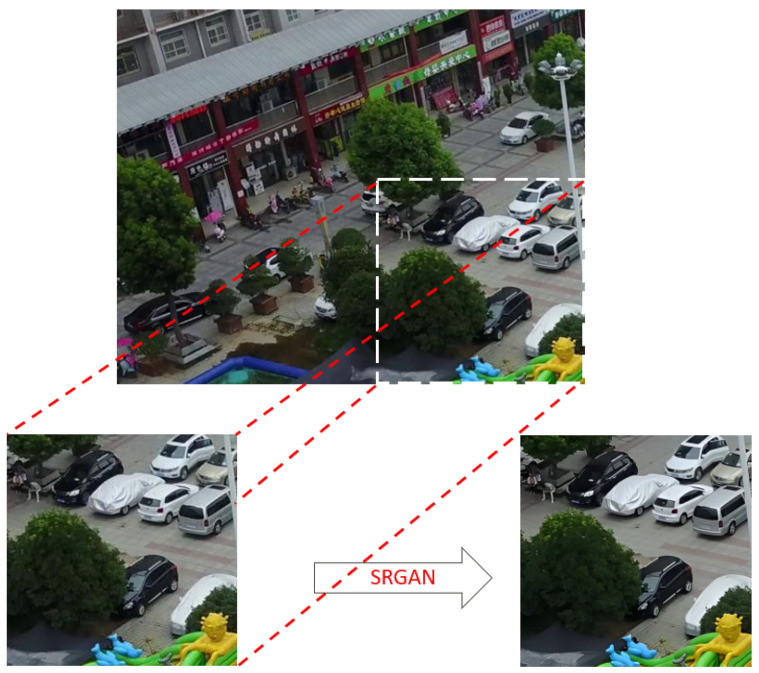
An example of the resolution change in the image after passing through the SRGAN. layer.

Here, *y* (*i*, *j*) represents the *i*, *j* pixel of the upsampled image; *x* represents the input image; and *s* represents the upsampling factor. The final layers of the model are used to obtain the high-resolution image. These layers consist of convolutional layers and activation functions. The parameters emerging from the last layer are input for the YOLO algorithm. The difference between the image passed through SRGAN layers and the original image can be seen in Figure 4.

**Figure 5:**
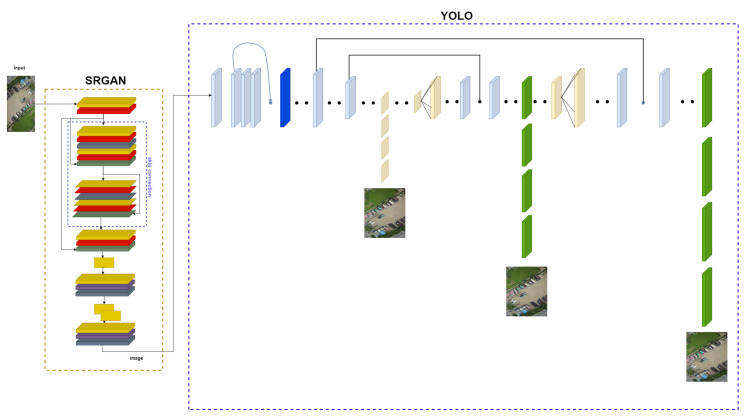
Block diagram of the overall structure of the SROD architecture.

The architecture of our proposed SROD algorithm can be seen in detail in Figure 5. This architecture is designed to optimize the integration of SRGAN and YOLO. High-resolution images produced by the SRGAN block are used as input to the YOLO architecture, making object detection more precise and reliable. As seen in Figure 5, the SRGAN block is now a layer of the YOLO architecture. The YOLO algorithm is preferred for object detection because it can process the image in one go. YOLO divides an image into grid cells and detects objects in each cell. This process is accomplished with a single feedforward pass, making the process extremely fast. This feature of YOLO makes it ideal for real-time applications. Additionally, YOLO’s ability to analyze the entire image simultaneously allows it to detect locations and classes of objects more accurately. Therefore, when used with SRGAN, YOLO significantly improves object detection performance.

## 4. Experimental Results and Discussion

The experimental studies using the proposed system obtained improved results compared to those of the existing methods. The existing object detection algorithms are shown with comparative tables that prevent pixel losses during image resizing, and optimizing the data labels increased the model performance. A detailed examination of the results revealed that pixel losses and errors in the labels directly affect the model performance. In order to test the performances of the proposed architecture, experiments were carried out on four different architectures of the YOLO algorithm. These architectures are YOLOv5 [30], YOLOv7 [31], YOLOv8 [32], and YOLOv9 [33], which are the most current and popular for object detection. Each architecture has the potential to show superior performance in specific object detection tasks, and the aim was to determine the performance increase with SRGAN integration. In order to compare the results of the proposed architecture with YOLO and to see its performance contribution, training was also carried out with original YOLO algorithms without SRGAN integrated, and the test results were examined on datasets. This comparison is of critical importance to see the impact of SRGAN. Testing without SRGAN integration helped determine the baseline performance level and more clearly demonstrated the contribution of the integration.

More objective results were achieved during the training by using standard hyperparameters in all YOLOv5, YOLOv7, YOLOv8, and YOLOv9 architectures. This approach allowed us to evaluate the performance of each architecture fairly. Using standard hyperparameters made comparisons between SROD and the original YOLO architectures more reliable and valid so that the real effects of SRGAN integration could be observed. The test results of the experimental studies on the xView and VisDrone datasets can be seen in Table 3 and Table 4, respectively.

In the experiments on the xView dataset, better mAP results were obtained compared to those of other models when tests were performed on images that had never been used with the YOLOv5 algorithm training weights. In the experiments conducted on the VisDrone dataset, better mAP results were obtained compared to those of other models when tests were performed on images that had never been used with the YOLOv8 algorithm training weights.

**Figure 6:**
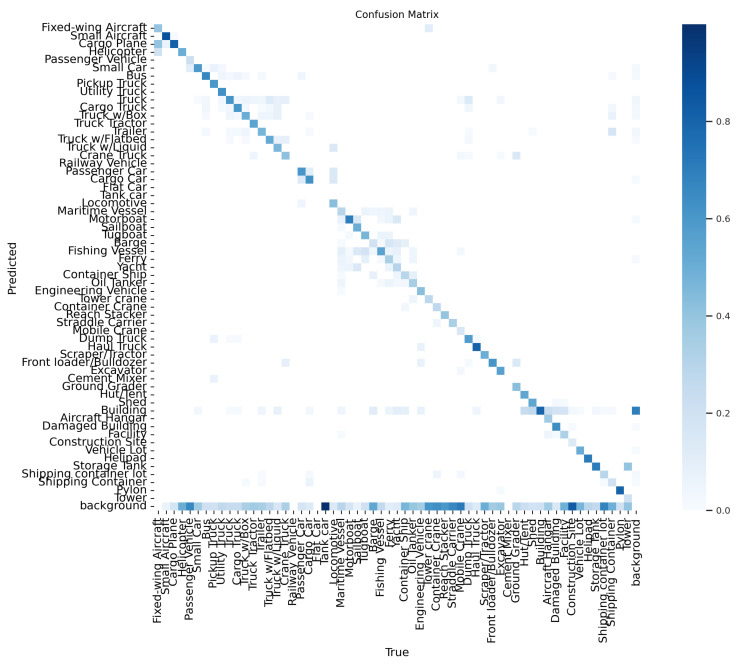
YOLOv5 confusion matrices, respectively, with the proposed method for xView datasets.

**Figure 7:**
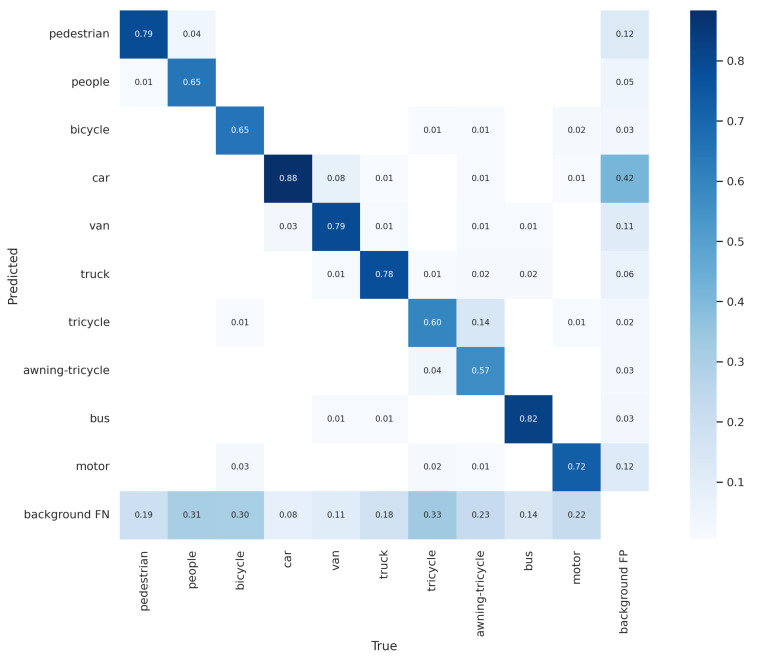
YOLOv8 confusion matrices, respectively, with the proposed method for VisDrone datasets.

A confusion matrix could be used to evaluate the performance of the trained model following tests using test data. The performance of a classification model is summarized in a confusion matrix table. The table contains predicted and actual class definitions and the proportion of correct and incorrect predictions for each class. AP belonging to classes is examined here, and it determines which classes are mixed together more. Hyperparameters of the models can be adjusted according to the examinations. This study obtained the best results of the xView and VisDrone datasets with YOLOv5 and YOLOv8, respectively. The corresponding confusion matrices are shown in Figure 6 and Figure 7. The diagonal members of the matrix represent the number of correct predictions (true positives) for each class. In contrast, the number of incorrect predictions is represented by the off-diagonal components (false positives and false negatives).

The results of the proposed system for VisDrone and xView datasets were better than those of the studies in the literature. The examination results are given in Figure 5. Among the existing studies, we compared the ones that achieved the best performance for xView and VisDrone datasets. As can be seen in Table 5, we achieved more successful mAP compared to studies in the literature.

When the test results in the study were examined, the pre-processing we proposed and the performance of the SROD algorithm, which is a hybrid model, gave remarkable results. The SROD algorithm increased the accuracy and efficiency of the model when used with various pre-processing applied to datasets. One of the most apparent advantages of the proposed SROD algorithm is its superior performance on high-difficulty datasets. These datasets contain vast numbers of small objects. The SROD algorithm produced consistent and reliable results even on these challenging datasets. This success is based on the algorithm’s flexibility and powerful modeling capacity.

The integration of SRGAN into YOLO increased the total computational cost. However, performance analysis and experimental results show that the increase in computational cost can be ignored. Despite the increase in computational cost, SRGAN’s super-resolution capability and YOLO’s effectiveness in object detection led to significant performance improvements, especially in high-resolution and complex images. In the literature, studies have been conducted in lightweight object detection where computational costs are low. The study conducted by Zhang et al. [34] examined the effectiveness of lightweight neural networks in object detection. In the study, it was shown that lightweight models can make faster and more efficient detections without sacrificing performance. In our next study, it is planned to minimize the computational costs along with the performance increase by integrating the lightweight neural network conducted by Zhang et al. [34] into the proposed SROD structure.

## 5. Conclusions

This study presents an efficient multi-staged method to improve the identification and localization of objects within images captured from high altitudes, such as satellite and aerial images. The pre-processing steps and SROD algorithm in the proposed method can be easily used in object detection studies on different types of images. The proposed method is designed to exhibit consistent performance without changing according to data types and sizes. Pre-processing steps are designed to be adjustable for different images. The SROD algorithm allows for the super-resolution enhancement of low-resolution images, leading to more precise object detection.

The proposed system contains multiple stages: ISA for pre-processing, SAM for checking objects in bounding boxes, and YOLO with the SRGAN for object detection. In order to test the proposed system, the xView and VisDrone datasets, widely used benchmarks for object detection and recognition tasks, were used. According to experiments, the proposed system significantly improved mAP, precision, and recall metrics performance.

Although the study focused on satellite and aerial imagery, the findings can be applied to other fields of object detection that use high-resolution images, such as medical imaging, autonomous driving, wildlife monitoring, and industrial inspection. The proposed system can improve similar applications that demand accurate object recognition. The components of the multi-staged structure, like SRGAN and YOLO, can further be improved to improve performance metrics.

## Figures and Tables

**Table 1:** General structure of xView dataset.

Feature	Explanation
Scope	Various geographical regions and environmental conditions around the world
Resolution	Very clear images where each pixel represents 30 cm
Labeling	More than 1 million tagging
Object Classes	More than 60 object classes
Applications	Military, urban planning, disaster management, environmental monitoring

**Table 2:** General structure of VisDrone dataset.

Feature	Explanation
Scope and diversity	Images taken under various weather conditions, lighting conditions, and environmental conditions
Image quality and resolution	High-resolution and detailed images
Labeling	Hundreds of thousands of labeled examples for more than 10 object classes
Tasks	Object detection, object tracking, and video analysis
Applications	Security monitoring, traffic management, disaster response, and urban planning

**Table 3:** xView dataset experimental test results.

Models	Precision	Recall	mAP_0.5	mAP_0.5:0.95	F1-Score
ISA-YOLOv5	59.9	44.5	45.6	30.1	52.1
ISA-SAM-YOLOv5	60.5	46.3	48.1	33.1	53.2
**ISA-SAM-SROD-YOLOv5**	**64.3**	**52.5**	**54.4**	**46.1**	**58.3**
ISA-YOLOv7	42.4	25.2	28.3	17.4	35.9
ISA-SAM-YOLOv7	48.2	27.9	30.3	21.1	37.8
ISA-SAM-SROD-YOLOv7	52.9	32.5	35.7	23.6	42.9
ISA-YOLOv8	53.7	31.2	33.5	22.6	42.1
ISA-SAM-YOLOv8	54.0	35.3	36.2	24.1	44.0
ISA-SAM-SROD-YOLOv8	61.4	39.8	41.2	28.5	54.3
ISA-YOLOv9	45.3	27.1	27.9	17.1	35.9
ISA-SAM-YOLOv9	46.3	28.6	29.8	20.0	37.2
ISA-SAM-SROD-YOLOv9	50.5	30.9	33.6	21.9	40.7

**Table 4:** VisDrone dataset experimental test results.

Models	Precision	Recall	mAP_0.5	mAP_0.5:0.95	F1-Score
ISA-YOLOv5	65.5	55.3	53.1	39.9	59.7
ISA-SAM-YOLOv5	67.3	56.4	55.8	43.2	61.7
ISA-SAM-SROD-YOLOv5	71.9	65.5	67.7	49.1	68.3
ISA-YOLOv7	68.1	64.8	67.4	44.0	66.4
ISA-SAM-YOLOv7	76.3	68.5	71.8	47.9	72.4
ISA-SAM-SROD-YOLOv7	81.2	70.6	75.6	53.5	75.9
ISA-YOLOv8	74.9	64.9	66.5	52.8	69.7
ISA-SAM-YOLOv8	80.1	61.4	69.6	55.4	70.6
**ISA-SAM-SROD-YOLOv8**	**80.4**	**74.4**	**77.5**	**63.8**	**77.9**
ISA-YOLOv9	59.3	45.8	47.8	35.3	51.9
ISA-SAM-YOLOv9	67.1	54.8	59.4	42.7	60.7
ISA-SAM-SROD-YOLOv9	71.6	63.2	65.1	48.7	67.2

**Table 5:** Performance comparison of the proposed method (* mean average recall (mAR)).

Model Name	Datasets	Number of Classes	mAP_0.5	mAP_0.5:0.95
Akyon et al. [13]	VisDrone	10	66.4	42.2
Akshatha et al. [19]	VisDrone	10	58.3	
Muzammul et al. [22]	VisDrone	10	73.7	54.8
**ISA-SAM-SROD-YOLOv8**	**VisDrone**	**10**	**77.5**	**63.8**
Akyon et al. [13]	xView	60	23.6	14.9
Olamofe et al. [14]	xView	6	44.7	* (mAR) 65.9
**ISA-SAM-SROD-YOLOv5**	**xView**	**60**	**54.4**	**46.1**

## Data Availability

The data required to reproduce the above findings are available to download from Scopus Document Searcher using the keywords specified at the methodology.
